# Tetra­aqua­(1,10-phenanthroline)zinc(II) 3,6-dicarboxy­bicyclo­[2.2.2]oct-7-ene-2,5-dicarboxyl­ate

**DOI:** 10.1107/S1600536808025622

**Published:** 2008-08-13

**Authors:** Chun-Hui Yu

**Affiliations:** a Department of Chemistry, College of Chemistry and Biology, Beihua University, Jilin City 132013, People’s Republic of China

## Abstract

In the title compound, [Zn(C_12_H_8_N_2_)(H_2_O)_4_](C_12_H_10_O_8_), each Zn^II^ atom is six-coordinated by two N atoms from one phenanthroline mol­ecule and by four O atoms from four water mol­ecules in a distorted octa­hedral environment. In the crystal structure, ions are linked by O—H⋯O hydrogen bonds.

## Related literature

For related literature, see: Ma *et al.* (2003 ▶); Hu (2008 ▶).
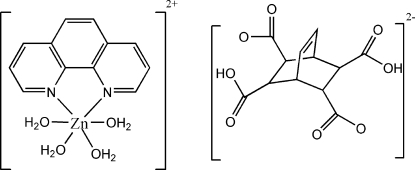


## Experimental

### 

#### Crystal data

[Zn(C_12_H_8_N_2_)(H_2_O)_4_](C_12_H_10_O_8_)
*M* *_r_* = 599.84
Monoclinic, 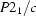

*a* = 7.4550 (2) Å
*b* = 13.5991 (4) Å
*c* = 22.9833 (7) Å
β = 91.555 (1)°
*V* = 2329.22 (12) Å^3^
*Z* = 4
Mo *K*α radiation
μ = 1.13 mm^−1^
*T* = 293 (2) K
0.33 × 0.22 × 0.19 mm

#### Data collection

Bruker APEX CCD area-detector diffractometer
Absorption correction: multi-scan (*SAINT*; Bruker, 1998 ▶) *T*_min_ = 0.687, *T*_max_ = 0.805
14301 measured reflections
5626 independent reflections
3854 reflections with *I* > 2σ(*I*)
*R*_int_ = 0.048

#### Refinement

*R*[*F* ^2^ > 2σ(*F* ^2^)] = 0.046
*wR*(*F* ^2^) = 0.122
*S* = 0.96
5626 reflections
382 parameters
12 restraints
H atoms treated by a mixture of independent and constrained refinement
Δρ_max_ = 0.50 e Å^−3^
Δρ_min_ = −0.59 e Å^−3^

### 

Data collection: *SMART* (Bruker, 1998 ▶); cell refinement: *SAINT* (Bruker, 1998 ▶); data reduction: *SAINT*; program(s) used to solve structure: *SHELXS97* (Sheldrick, 2008 ▶); program(s) used to refine structure: *SHELXL97* (Sheldrick, 2008 ▶); molecular graphics: *SHELXTL* (Sheldrick, 2008 ▶); software used to prepare material for publication: *SHELXTL*.

## Supplementary Material

Crystal structure: contains datablocks global, I. DOI: 10.1107/S1600536808025622/bt2766sup1.cif

Structure factors: contains datablocks I. DOI: 10.1107/S1600536808025622/bt2766Isup2.hkl

Additional supplementary materials:  crystallographic information; 3D view; checkCIF report

## Figures and Tables

**Table 1:** Hydrogen-bond geometry (Å, °)

*D*—H⋯*A*	*D*—H	H⋯*A*	*D*⋯*A*	*D*—H⋯*A*
O3—H3⋯O8^i^	0.82	1.88	2.674 (3)	163
O6—H6*A*⋯O2^ii^	0.82	1.84	2.583 (3)	151
O2*W*—H*W*22⋯O2^iii^	0.799 (17)	2.06 (2)	2.818 (3)	158 (4)
O4*W*—H*W*41⋯O1	0.845 (18)	1.86 (2)	2.705 (3)	174 (4)
O2*W*—H*W*21⋯O8^iv^	0.809 (17)	2.121 (18)	2.904 (3)	163 (4)
O4*W*—H*W*42⋯O7^iv^	0.811 (17)	1.876 (17)	2.686 (3)	177 (3)
O1*W*—H*W*12⋯O7^v^	0.816 (17)	2.09 (2)	2.884 (3)	165 (3)
O1*W*—H*W*11⋯O2^vi^	0.826 (18)	2.13 (2)	2.923 (3)	160 (4)
O3*W*—H*W*31⋯O2^vi^	0.80 (4)	2.57 (4)	3.233 (4)	142 (5)

Symmetry codes: (i) 
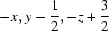
; (ii) 
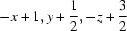
; (iii) 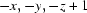; (iv) 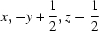; (v) 
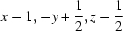
; (vi) 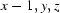.

**Fig. 1.**
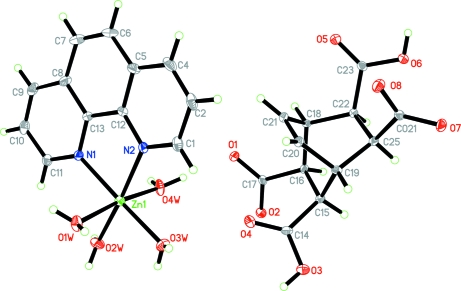
The structure of the title compound, showing the atomic numbering scheme. Displacement ellipsoids are drawn at the 30% probability level.

## supplementary crystallographic information

### Comment

Recently, complexes with poly(carboxylic acids) have been investigated in the area of solid state and material science (Ma *et al.*, 2003). I selected bicyclo[2.2.2]oct-7-ene-2,3,5,6-tetracarboxylic acid (H_4_L) as a poly(carboxylic acid) ligand and phenanthroline (phen) as a secondary ligand, generating a new complex, [Zn(phen)(H_2_O)_4_]^.^[H_2_L],   which is reported here.

In the title compound, [Zn(phen)(H_2_O)_4_]^.^[H_2_L], each Zn^II^ atom is six-coordinated by two N atoms from one phen molecule, and four O atoms from four water molecules in a distorted octahedral environment. Each H_2_*L*^2-^ acts as a counter-anion. In the crystal, the molecules are linked by O—H···O hydrogen bonds.

### Experimental

A mixture of H_4_L (0.5 mmol), phen (0.5 mmol), NaOH (1 mmol) and ZnCl_2_^.^6H_2_O (0.5 mmol) was suspended in 12 ml of deionized water and sealed in a 20-ml Teflon-lined autoclave. Upon heating at 140°C for one week, the autoclave was slowly cooled to room temperature. The crystals were collected, washed with deionized water and dried.

### Refinement

All H atoms on C atoms were positioned geometrically (C—H = 0.93 Å) and refined as riding, with *U*_iso_(H)=1.2*U*_eq_(carrier). The water H-atoms were located in a difference Fourier map, and were refined with distance restraints of O–H = 0.85±0.01 Å; their displacement parameters were set to 1.2U_eq_(O).

### Crystal data

**Table:**

[Zn(C_12_H_8_N_2_)(H_2_O)_4_](C_12_H_10_O_8_)	*F*_000_ = 1240
*M**_r_* = 599.84	*D*_x_ = 1.711 Mg m^−^^3^
Monoclinic, *P*2_1_/*c*	Mo *K*α radiation λ = 0.71073 Å
Hall symbol: -P 2ybc	Cell parameters from 5626 reflections
*a* = 7.4550 (2) Å	θ = 1.1–28.3º
*b* = 13.5991 (4) Å	µ = 1.13 mm^−^^1^
*c* = 22.9833 (7) Å	*T* = 293 (2) K
β = 91.5550 (10)º	Block, colorless
*V* = 2329.22 (12) Å^3^	0.33 × 0.22 × 0.19 mm
*Z* = 4	

### Data collection

**Table:**

Bruker APEX CCD area-detector diffractometer	5626 independent reflections
Radiation source: fine-focus sealed tube	3854 reflections with *I* > 2σ(*I*)
Monochromator: graphite	*R*_int_ = 0.048
*T* = 293(2) K	θ_max_ = 28.3º
φ and ω scans	θ_min_ = 1.7º
Absorption correction: multi-scan(SAINT; Bruker, 1998)	*h* = −9→8
*T*_min_ = 0.687, *T*_max_ = 0.805	*k* = −14→18
14301 measured reflections	*l* = −29→30

### Refinement

**Table:**

Refinement on *F*^2^	Secondary atom site location: difference Fourier map
Least-squares matrix: full	Hydrogen site location: inferred from neighbouring sites
*R*[*F*^2^ > 2σ(*F*^2^)] = 0.046	H atoms treated by a mixture of independent and constrained refinement
*wR*(*F*^2^) = 0.122	*w* = 1/[σ^2^(*F*_o_^2^) + (0.0613*P*)^2^] where *P* = (*F*_o_^2^ + 2*F*_c_^2^)/3
*S* = 0.96	(Δ/σ)_max_ < 0.001
5626 reflections	Δρ_max_ = 0.50 e Å^−^^3^
382 parameters	Δρ_min_ = −0.59 e Å^−^^3^
12 restraints	Extinction correction: none
Primary atom site location: structure-invariant direct methods	

### Special details

**Table:**

Geometry. All e.s.d.'s (except the e.s.d. in the dihedral angle between two l.s. planes) are estimated using the full covariance matrix. The cell e.s.d.'s are taken into account individually in the estimation of e.s.d.'s in distances, angles and torsion angles; correlations between e.s.d.'s in cell parameters are only used when they are defined by crystal symmetry. An approximate (isotropic) treatment of cell e.s.d.'s is used for estimating e.s.d.'s involving l.s. planes.
Refinement. Refinement of *F*^2^ against ALL reflections. The weighted *R*-factor *wR* and goodness of fit *S* are based on *F*^2^, conventional *R*-factors *R* are based on *F*, with *F* set to zero for negative *F*^2^. The threshold expression of *F*^2^ > σ(*F*^2^) is used only for calculating *R*-factors(gt) *etc*. and is not relevant to the choice of reflections for refinement. *R*-factors based on *F*^2^ are statistically about twice as large as those based on *F*, and *R*- factors based on ALL data will be even larger.

### Fractional atomic coordinates and isotropic or equivalent isotropic displacement parameters (Å^2^)

**Table:**

	*x*	*y*	*z*	*U*_iso_*/*U*_eq_	
C1	−0.2556 (4)	0.2855 (2)	0.61401 (12)	0.0348 (7)	
H1	−0.2529	0.2264	0.6344	0.042*	
C2	−0.2659 (4)	0.3745 (3)	0.64532 (13)	0.0414 (8)	
H2	−0.2708	0.3734	0.6857	0.050*	
C4	−0.2686 (5)	0.4609 (3)	0.61680 (15)	0.0459 (9)	
H4	−0.2756	0.5194	0.6376	0.055*	
C5	−0.2610 (5)	0.4632 (2)	0.55588 (14)	0.0399 (8)	
C6	−0.2629 (6)	0.5510 (2)	0.52184 (18)	0.0570 (10)	
H6	−0.2628	0.6117	0.5405	0.068*	
C7	−0.2648 (6)	0.5479 (2)	0.46388 (18)	0.0554 (10)	
H7	−0.2692	0.6064	0.4430	0.067*	
C8	−0.2603 (5)	0.4564 (2)	0.43289 (15)	0.0385 (8)	
C9	−0.2666 (4)	0.4472 (2)	0.37212 (14)	0.0409 (8)	
H9	−0.2727	0.5032	0.3489	0.049*	
C10	−0.2639 (4)	0.3579 (2)	0.34720 (13)	0.0377 (8)	
H10	−0.2694	0.3523	0.3069	0.045*	
C11	−0.2527 (4)	0.2731 (2)	0.38191 (12)	0.0307 (7)	
H11	−0.2502	0.2118	0.3641	0.037*	
C12	−0.2538 (4)	0.3703 (2)	0.52783 (12)	0.0272 (6)	
C13	−0.2518 (4)	0.3674 (2)	0.46512 (12)	0.0271 (6)	
C14	0.1068 (4)	0.06219 (19)	0.70078 (12)	0.0268 (6)	
C15	0.2475 (4)	0.12469 (19)	0.73318 (11)	0.0220 (6)	
H15	0.3295	0.0808	0.7548	0.026*	
C16	0.3605 (4)	0.19231 (18)	0.69329 (10)	0.0206 (5)	
H16	0.4869	0.1784	0.7027	0.025*	
C17	0.3320 (4)	0.1720 (2)	0.62817 (11)	0.0252 (6)	
C18	0.3279 (3)	0.30060 (18)	0.70964 (10)	0.0187 (5)	
H18	0.3894	0.3445	0.6829	0.022*	
C19	0.1500 (4)	0.19201 (18)	0.77770 (11)	0.0209 (5)	
H19	0.0744	0.1524	0.8029	0.025*	
C20	0.0380 (4)	0.26442 (19)	0.74361 (11)	0.0243 (6)	
H20	−0.0857	0.2692	0.7469	0.029*	
C021	0.2081 (4)	0.3018 (2)	0.86562 (11)	0.0234 (6)	
C21	0.1303 (4)	0.32112 (19)	0.70833 (11)	0.0245 (6)	
H21	0.0773	0.3693	0.6849	0.029*	
C22	0.4016 (4)	0.31473 (18)	0.77246 (11)	0.0211 (5)	
H22	0.5246	0.2892	0.7732	0.025*	
C23	0.4151 (4)	0.4227 (2)	0.79029 (11)	0.0262 (6)	
C25	0.2936 (4)	0.24790 (18)	0.81443 (10)	0.0216 (6)	
H25	0.3771	0.1990	0.8309	0.026*	
N1	−0.2458 (3)	0.27831 (16)	0.43985 (9)	0.0246 (5)	
N2	−0.2495 (3)	0.28378 (17)	0.55612 (9)	0.0255 (5)	
O1	0.2699 (3)	0.23710 (15)	0.59560 (8)	0.0417 (6)	
O2	0.3860 (3)	0.08866 (14)	0.60983 (8)	0.0348 (5)	
O1W	−0.4920 (3)	0.13197 (19)	0.49279 (10)	0.0375 (5)	
O3	0.1019 (3)	−0.02720 (15)	0.72076 (9)	0.0469 (6)	
H3	0.0255	−0.0587	0.7024	0.070*	
O2W	−0.1668 (4)	0.06205 (18)	0.43708 (11)	0.0422 (6)	
O4	0.0122 (3)	0.09170 (16)	0.66157 (10)	0.0473 (6)	
O3W	−0.2116 (4)	0.0585 (2)	0.56396 (12)	0.0490 (6)	
O5	0.3699 (3)	0.49114 (14)	0.76039 (9)	0.0399 (5)	
O4W	0.0517 (3)	0.17941 (19)	0.50606 (9)	0.0401 (6)	
O6	0.4927 (3)	0.42964 (15)	0.84189 (8)	0.0374 (5)	
H6A	0.4995	0.4877	0.8514	0.056*	
O7	0.2531 (3)	0.27139 (14)	0.91541 (8)	0.0336 (5)	
O8	0.0968 (3)	0.36877 (15)	0.85579 (8)	0.0371 (5)	
Zn1	−0.22063 (5)	0.16487 (2)	0.499735 (13)	0.02916 (12)	
HW22	−0.223 (4)	0.013 (2)	0.4325 (16)	0.063 (13)*	
HW41	0.113 (5)	0.198 (3)	0.5355 (10)	0.095*	
HW21	−0.092 (4)	0.069 (3)	0.4124 (14)	0.074 (15)*	
HW42	0.109 (4)	0.195 (2)	0.4781 (9)	0.037 (10)*	
HW12	−0.551 (4)	0.168 (2)	0.4714 (11)	0.043 (11)*	
HW11	−0.551 (5)	0.118 (3)	0.5214 (12)	0.079 (15)*	
HW31	−0.282 (6)	0.057 (3)	0.5893 (17)	0.119*	
HW32	−0.195 (6)	0.009 (2)	0.5475 (17)	0.083 (18)*	

### Atomic displacement parameters (Å^2^)

**Table:**

	*U*^11^	*U*^22^	*U*^33^	*U*^12^	*U*^13^	*U*^23^
C1	0.0317 (17)	0.049 (2)	0.0242 (14)	0.0001 (14)	0.0003 (12)	−0.0013 (14)
C2	0.0318 (18)	0.067 (2)	0.0255 (15)	0.0106 (16)	−0.0019 (13)	−0.0162 (16)
C4	0.043 (2)	0.049 (2)	0.0447 (19)	0.0084 (16)	−0.0065 (16)	−0.0268 (17)
C5	0.045 (2)	0.0296 (17)	0.0447 (19)	0.0040 (14)	−0.0066 (15)	−0.0126 (15)
C6	0.079 (3)	0.0198 (17)	0.072 (3)	0.0021 (16)	−0.007 (2)	−0.0094 (18)
C7	0.074 (3)	0.0221 (18)	0.070 (3)	0.0012 (17)	−0.004 (2)	0.0069 (17)
C8	0.044 (2)	0.0230 (16)	0.0478 (19)	−0.0010 (13)	−0.0031 (15)	0.0094 (14)
C9	0.041 (2)	0.0408 (19)	0.0404 (18)	−0.0060 (15)	−0.0033 (15)	0.0234 (15)
C10	0.0336 (18)	0.054 (2)	0.0251 (15)	0.0009 (14)	0.0010 (13)	0.0107 (15)
C11	0.0318 (16)	0.0369 (17)	0.0235 (14)	0.0024 (13)	−0.0001 (12)	0.0001 (13)
C12	0.0309 (16)	0.0223 (14)	0.0283 (15)	−0.0003 (11)	−0.0022 (12)	−0.0038 (12)
C13	0.0319 (16)	0.0208 (14)	0.0283 (14)	−0.0005 (11)	−0.0027 (12)	0.0022 (12)
C14	0.0353 (17)	0.0180 (14)	0.0273 (14)	−0.0034 (11)	0.0054 (13)	−0.0008 (11)
C15	0.0283 (15)	0.0176 (13)	0.0201 (13)	−0.0006 (10)	0.0004 (11)	0.0004 (10)
C16	0.0236 (14)	0.0201 (13)	0.0180 (12)	0.0012 (10)	0.0012 (11)	0.0023 (10)
C17	0.0331 (16)	0.0264 (15)	0.0161 (12)	−0.0042 (12)	−0.0006 (11)	−0.0004 (11)
C18	0.0241 (14)	0.0152 (12)	0.0167 (12)	−0.0037 (10)	0.0017 (10)	0.0019 (10)
C19	0.0234 (14)	0.0178 (13)	0.0218 (13)	−0.0028 (10)	0.0054 (11)	0.0012 (10)
C20	0.0220 (14)	0.0252 (14)	0.0255 (14)	0.0014 (11)	−0.0029 (11)	−0.0002 (11)
C021	0.0296 (15)	0.0219 (14)	0.0188 (13)	−0.0019 (11)	0.0028 (11)	−0.0009 (11)
C21	0.0279 (15)	0.0220 (14)	0.0233 (13)	0.0030 (11)	−0.0051 (11)	0.0010 (11)
C22	0.0233 (14)	0.0196 (13)	0.0203 (13)	0.0002 (10)	−0.0001 (11)	0.0007 (10)
C23	0.0341 (16)	0.0241 (15)	0.0204 (13)	−0.0099 (12)	0.0031 (12)	−0.0014 (11)
C25	0.0248 (14)	0.0187 (13)	0.0213 (13)	0.0027 (10)	0.0006 (11)	0.0001 (11)
N1	0.0286 (13)	0.0241 (12)	0.0212 (11)	0.0011 (10)	−0.0006 (9)	0.0011 (10)
N2	0.0282 (13)	0.0278 (13)	0.0203 (11)	0.0000 (10)	−0.0019 (9)	−0.0015 (10)
O1	0.0729 (17)	0.0249 (11)	0.0264 (10)	−0.0026 (10)	−0.0152 (11)	0.0063 (9)
O2	0.0529 (14)	0.0266 (11)	0.0248 (10)	0.0048 (9)	−0.0025 (10)	−0.0068 (9)
O1W	0.0282 (12)	0.0574 (16)	0.0270 (12)	−0.0047 (11)	0.0014 (10)	0.0052 (11)
O3	0.0640 (17)	0.0305 (12)	0.0454 (13)	−0.0204 (11)	−0.0111 (12)	0.0083 (10)
O2W	0.0487 (16)	0.0325 (14)	0.0457 (14)	−0.0005 (11)	0.0034 (12)	−0.0171 (11)
O4	0.0527 (15)	0.0370 (13)	0.0507 (14)	−0.0078 (11)	−0.0267 (12)	0.0000 (11)
O3W	0.0611 (18)	0.0372 (15)	0.0482 (15)	−0.0090 (12)	−0.0082 (13)	0.0157 (13)
O5	0.0610 (16)	0.0250 (11)	0.0330 (11)	−0.0052 (10)	−0.0117 (10)	0.0037 (9)
O4W	0.0259 (12)	0.0736 (18)	0.0208 (10)	−0.0078 (11)	0.0007 (9)	0.0006 (11)
O6	0.0551 (15)	0.0235 (11)	0.0328 (11)	−0.0068 (9)	−0.0142 (10)	−0.0010 (9)
O7	0.0495 (14)	0.0329 (12)	0.0184 (9)	0.0085 (10)	0.0031 (9)	0.0025 (8)
O8	0.0507 (14)	0.0341 (12)	0.0267 (11)	0.0174 (10)	0.0063 (10)	0.0014 (9)
Zn1	0.0328 (2)	0.0265 (2)	0.0280 (2)	0.00085 (14)	−0.00117 (14)	0.00085 (14)

### Geometric parameters (Å, °)

**Table:**

C1—N2	1.333 (3)	C18—C21	1.499 (4)
C1—C2	1.410 (4)	C18—C22	1.543 (3)
C1—H1	0.9300	C18—H18	0.9800
C2—C4	1.346 (5)	C19—C20	1.498 (4)
C2—H2	0.9300	C19—C25	1.545 (4)
C4—C5	1.403 (5)	C19—H19	0.9800
C4—H4	0.9300	C20—C21	1.325 (4)
C5—C12	1.420 (4)	C20—H20	0.9300
C5—C6	1.428 (5)	C021—O8	1.248 (3)
C6—C7	1.333 (6)	C021—O7	1.254 (3)
C6—H6	0.9300	C021—C25	1.539 (3)
C7—C8	1.434 (5)	C21—H21	0.9300
C7—H7	0.9300	C22—C23	1.526 (3)
C8—C9	1.402 (4)	C22—C25	1.564 (3)
C8—C13	1.419 (4)	C22—H22	0.9800
C9—C10	1.343 (4)	C23—O5	1.200 (3)
C9—H9	0.9300	C23—O6	1.309 (3)
C10—C11	1.403 (4)	C25—H25	0.9800
C10—H10	0.9300	N1—Zn1	2.073 (2)
C11—N1	1.333 (3)	N2—Zn1	2.087 (2)
C11—H11	0.9300	O1W—Zn1	2.074 (2)
C12—N2	1.344 (3)	O1W—HW12	0.816 (17)
C12—C13	1.442 (4)	O1W—HW11	0.826 (18)
C13—N1	1.345 (3)	O3—H3	0.8200
C14—O4	1.198 (3)	O2W—Zn1	2.055 (2)
C14—O3	1.300 (3)	O2W—HW22	0.799 (17)
C14—C15	1.528 (4)	O2W—HW21	0.809 (17)
C15—C16	1.561 (3)	O3W—Zn1	2.067 (2)
C15—C19	1.567 (3)	O3W—HW31	0.80 (4)
C15—H15	0.9800	O3W—HW32	0.784 (18)
C16—C17	1.531 (3)	O4W—Zn1	2.041 (2)
C16—C18	1.541 (3)	O4W—HW41	0.845 (18)
C16—H16	0.9800	O4W—HW42	0.811 (17)
C17—O1	1.240 (3)	O6—H6A	0.8200
C17—O2	1.279 (3)		
			
N2—C1—C2	121.9 (3)	C20—C19—H19	110.4
N2—C1—H1	119.1	C25—C19—H19	110.4
C2—C1—H1	119.1	C15—C19—H19	110.4
C4—C2—C1	120.0 (3)	C21—C20—C19	114.3 (2)
C4—C2—H2	120.0	C21—C20—H20	122.9
C1—C2—H2	120.0	C19—C20—H20	122.9
C2—C4—C5	120.3 (3)	O8—C021—O7	124.5 (2)
C2—C4—H4	119.8	O8—C021—C25	119.8 (2)
C5—C4—H4	119.8	O7—C021—C25	115.7 (2)
C4—C5—C12	115.9 (3)	C20—C21—C18	113.9 (2)
C4—C5—C6	124.4 (3)	C20—C21—H21	123.0
C12—C5—C6	119.7 (3)	C18—C21—H21	123.0
C7—C6—C5	121.4 (3)	C23—C22—C18	113.0 (2)
C7—C6—H6	119.3	C23—C22—C25	115.1 (2)
C5—C6—H6	119.3	C18—C22—C25	109.2 (2)
C6—C7—C8	121.7 (3)	C23—C22—H22	106.3
C6—C7—H7	119.2	C18—C22—H22	106.3
C8—C7—H7	119.2	C25—C22—H22	106.3
C9—C8—C13	116.4 (3)	O5—C23—O6	124.9 (3)
C9—C8—C7	124.9 (3)	O5—C23—C22	125.2 (2)
C13—C8—C7	118.8 (3)	O6—C23—C22	109.7 (2)
C10—C9—C8	120.3 (3)	C021—C25—C19	110.9 (2)
C10—C9—H9	119.8	C021—C25—C22	115.2 (2)
C8—C9—H9	119.8	C19—C25—C22	108.0 (2)
C9—C10—C11	120.1 (3)	C021—C25—H25	107.5
C9—C10—H10	119.9	C19—C25—H25	107.5
C11—C10—H10	119.9	C22—C25—H25	107.5
N1—C11—C10	121.6 (3)	C11—N1—C13	118.6 (2)
N1—C11—H11	119.2	C11—N1—Zn1	128.64 (19)
C10—C11—H11	119.2	C13—N1—Zn1	112.79 (17)
N2—C12—C5	124.0 (3)	C1—N2—C12	117.8 (3)
N2—C12—C13	117.4 (2)	C1—N2—Zn1	129.8 (2)
C5—C12—C13	118.6 (3)	C12—N2—Zn1	112.28 (17)
N1—C13—C8	123.0 (3)	Zn1—O1W—HW12	115 (2)
N1—C13—C12	117.2 (2)	Zn1—O1W—HW11	122 (3)
C8—C13—C12	119.8 (3)	HW12—O1W—HW11	109 (3)
O4—C14—O3	123.8 (3)	C14—O3—H3	109.5
O4—C14—C15	124.2 (2)	Zn1—O2W—HW22	124 (3)
O3—C14—C15	111.9 (2)	Zn1—O2W—HW21	124 (3)
C14—C15—C16	114.5 (2)	HW22—O2W—HW21	112 (3)
C14—C15—C19	108.6 (2)	Zn1—O3W—HW31	122 (4)
C16—C15—C19	107.85 (19)	Zn1—O3W—HW32	105 (3)
C14—C15—H15	108.6	HW31—O3W—HW32	117 (3)
C16—C15—H15	108.6	Zn1—O4W—HW41	127 (3)
C19—C15—H15	108.6	Zn1—O4W—HW42	121 (2)
C17—C16—C18	113.1 (2)	HW41—O4W—HW42	106 (3)
C17—C16—C15	113.9 (2)	C23—O6—H6A	109.5
C18—C16—C15	109.2 (2)	O4W—Zn1—O2W	84.42 (10)
C17—C16—H16	106.7	O4W—Zn1—O3W	90.23 (10)
C18—C16—H16	106.7	O2W—Zn1—O3W	91.20 (12)
C15—C16—H16	106.7	O4W—Zn1—N1	92.69 (9)
O1—C17—O2	123.3 (2)	O2W—Zn1—N1	93.26 (10)
O1—C17—C16	120.1 (2)	O3W—Zn1—N1	174.89 (11)
O2—C17—C16	116.4 (2)	O4W—Zn1—O1W	173.09 (10)
C21—C18—C16	109.5 (2)	O2W—Zn1—O1W	90.46 (10)
C21—C18—C22	108.7 (2)	O3W—Zn1—O1W	85.22 (10)
C16—C18—C22	107.0 (2)	N1—Zn1—O1W	92.24 (9)
C21—C18—H18	110.5	O4W—Zn1—N2	90.00 (9)
C16—C18—H18	110.5	O2W—Zn1—N2	171.08 (10)
C22—C18—H18	110.5	O3W—Zn1—N2	95.79 (11)
C20—C19—C25	109.3 (2)	N1—Zn1—N2	80.02 (9)
C20—C19—C15	107.7 (2)	O1W—Zn1—N2	95.62 (9)
C25—C19—C15	108.5 (2)		

### Hydrogen-bond geometry (Å, °)

**Table:**

*D*—H···*A*	*D*—H	H···*A*	*D*···*A*	*D*—H···*A*
O3—H3···O8^i^	0.82	1.88	2.674 (3)	163
O6—H6A···O2^ii^	0.82	1.84	2.583 (3)	151
O2W—HW22···O2^iii^	0.799 (17)	2.06 (2)	2.818 (3)	158 (4)
O4W—HW41···O1	0.845 (18)	1.86 (2)	2.705 (3)	174 (4)
O2W—HW21···O8^iv^	0.809 (17)	2.121 (18)	2.904 (3)	163 (4)
O4W—HW42···O7^iv^	0.811 (17)	1.876 (17)	2.686 (3)	177 (3)
O1W—HW12···O7^v^	0.816 (17)	2.09 (2)	2.884 (3)	165 (3)
O1W—HW11···O2^vi^	0.826 (18)	2.13 (2)	2.923 (3)	160 (4)
O3W—HW31···O2^vi^	0.80 (4)	2.57 (4)	3.233 (4)	142 (5)

Symmetry codes: (i) −*x*, *y*−1/2, −*z*+3/2; (ii) −*x*+1, *y*+1/2, −*z*+3/2; (iii) −*x*, −*y*, −*z*+1; (iv) *x*, −*y*+1/2, *z*−1/2; (v) *x*−1, −*y*+1/2, *z*−1/2; (vi) *x*−1, *y*, *z*.
